# Fisetin and/or capecitabine causes changes in apoptosis pathways in capecitabine-resistant colorectal cancer cell lines

**DOI:** 10.1007/s00210-024-03145-0

**Published:** 2024-05-15

**Authors:** Kanli Zehra, Aydin Banu, Erzik Can, Cabadak Hülya

**Affiliations:** 1 Institute of Health Sciences, Marmara University, Basibuyuk-Maltepe, Istanbul, 34854 Turkey; 2 School of Medicine, Department of Biophysics, Marmara University, Basic Medical Sciences Building, Maltepe, Istanbul, 34854 Turkey; 3 School of Medicine, Department of Medical Biology, Marmara University, Basic Medical Sciences Building, Maltepe, Istanbul, 34854 Turkey

**Keywords:** Capecitabine-resistance, HT29 cell, Fisetin, Apoptosis, Wound-healing

## Abstract

Capecitabine is recommended as one of the first-line chemotherapy treatments for advanced or metastatic colorectal cancer. Researches have been conducted on capecitabine’s impact on the viability of human colon cancer cells and its potential to induce apoptosis. However, even in cases initially responsive to treatment, the development of acquired resistance significantly limits its efficacy. Challenges still exist in effectively treating patients with chemotherapy, and developing new cytotoxic drugs is hindered by drug resistance. Fisetin alters the cell cycle, inducing apoptosis, inhibiting cancer cell proliferation, and enhancing the therapeutic effectiveness of chemotherapy drugs. This work aims to create a plan for reversing capecitabine resistance. For this purpose, the role of capecitabine and/or fisetin combinations in cell proliferation and apoptosis has been determined in both wild-type and capecitabine-resistant HT29 cells (CR/HT29). We developed capecitabine-resistant cell line from wild-type HT29 cells. This study demonstrated the effects of capecitabine, fisetin, and their combinations on both resistant and wild-type cells through experiments including cell survival skills, cell proliferation, wound healing, colony formation, hoechst staining, and western blot analysis. We established capecitabine-resistant cell lines. P-gp expression increased in CR/HT29 cells. Capecitabine effects on a CR/HT29 cells less than wild-type HT29 cells. The combination of fisetin and capecitabine in cell proliferation caused greater reductions in wild-type HT29 cells than in capecitabine-resistant cells. Fisetin has also additive effects on the apoptotic pathway in CR/HT29 cells. This study provides new perspectives on the combination of capecitabine and/or flavonoid treatment in resistant cells.

**Graphical abstract:**

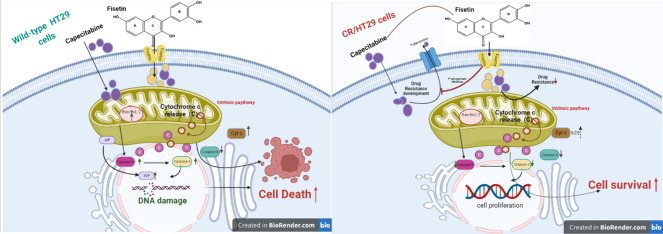

## Introduction

For years, colorectal cancer (CRC) has remained one of the most widespread ailments all around the world, resulting in extremely high death rates and is shown to be the third most mainly diagnosed and second most deadly cancer. However, research indicates that by 2035, the global incidence of colorectal cancer is anticipated to more than quadruple due to a large increase in occurrences among the older population (Hossain et al. 2022). During the development of colorectal adenocarcinomas, sequential genetic, and epigenetic mutations are observed in certain genes, leading to the initiation, progression, and metastasis of CRC (Brenner et al. 2014; Ewing et al. 2014). Despite the fact that increasing quantity of targeted medications accessible for the management of colorectal cancer, in terms of CRC patients, the 5-year survival rate is merely 65%. However, the therapeutic effect of chemotherapeutic drugs is often weakened by the acquired resistance of CRC cells during chemotherapeutic treatment. Thus, it is urgently necessary to shed light on the chemoresistance mechanisms and search for new optimal clinical strategies (Miller et al. 2016) Chemotherapy using a 5-fluorouracil (5-FU) regimen has been the mainstay of treatment for patients with colorectal cancer since the 1950s (Salonga et al. 2000). Drug treatments such as 5-FU, capecitabine, oxaliplatin, and irinotecan have been widely developed in colorectal cancer chemotherapy in recent decades, but unfortunately, the responses of these drugs and combined treatment remain confined (Yaffee et al. 2015). Capecitabine is a fluoropyrimidine that metaboılize to 5-FU in cancer cells. It is increasing the anticancer effects of 5-FU and achieving lower toxicity than 5-FU. It passes unchanged through the digestive tract and is subsequently converted to 5-deoxy-5-fluorocytidine (5′-DFCR) in the liver. Cytidine deaminase and carboxylesterase then convert capecitabine to doxyfluridine (5′-DFUR). Thymidine phosphorylase (TP) at the tumor site then finally converts 5′-DFUR to 5-FU (Di Costanzo et al. 2000). Even though histopathological and genetic factors in CRC have been extensively investigated, factors affecting the risk of disease recurrence in patients with different clinical outcomes are not fully understood (Stintzing et al. 2016). Some of these factors are responsible for drug resistance development. Although tumor cells initially respond to treatment, they can develop a resistance similar to classical acquired resistance. However, cancer cells the intrinsic or acquired resistance to capecitabine lead to disappointing results in CRC patients.

Multi-drug resistance (MDR) proteins mediate most drug resistance mediated by P-glycoprotein (P-gp) is a potent member of the MDR family and is essential in reducing therapeutic efficacy in most treatments. P-gp functions to safeguard cells by preventing the entry of dangerous substances including xenobiotics, but since it is overexpressed in cancerous cells, preventing access to the drug, its effect must be reduced in such cases for therapeutic efficacy (Estevinho et al. 2022).

Recently, plant compounds have begun to be used frequently in the treatment of drug resistance. Tetrahydroxyflavone, or fisetin, is a bioactive polyphenolic flavonoid that is primarily present in a wide range of fruits and vegetables, such as apples, strawberries, dates, onions, and cucumbers of different kinds (Suh et al. 2009).

In preclinical studies, it has been shown that fisetin decreases cancer cell proliferation, cell cycle alteretion, apoptosis, angiogenesis, invasion, and metastasis without causing any toxic effects on normal cells (Zhuo et al. 2015). Recent studies have shown that treatment with fisetin, in combination with oxaliplatin, significantly reduces cell proliferation of irinotecan-resistant LoVo cells compared to parental LoVo cancer cells (Jeng et al. 2018). Damaged cells undergo apoptosis in reaction to oncogene expression, cellular stress, or DNA damage. As a result of the destruction of damaged cells by apoptosis, healthy tissues and organs remain intact and growth of tumors is stopped (Pistritto et al. 2016). Additionally, apoptosis is necessary for protecting tissue homeostasis and regulating the functions of the immune system. All tumor cells aim to inhibit apoptotic pathways to survive. Cancer treatment protocols often target cell death pathways (Shirjang et al. 2019). Apoptotic signaling pathways are divided into two main classes: extrinsic and intrinsic pathways (Verbrugge et al. 2010). The intrinsic (or mitochondrial) apoptotic pathway is affected by various cellular stimuli such as DNA damage, cytotoxic drug therapy, lacking of growth factor, and oxidative stress. Members of the BcL-2 family (Bax, Bak, BcL-2, BcL-XL, MCL-1, Bid, and Bim) regulate the release of cytochrome c by controlling mitochondrial outer membrane permeability (Elmore 2007; White et al. 2003). AIF (apoptosis-inducing factor), localized in the mitochondrial membrane, is active in the mitochondrial apoptotic pathway, AIF moves from the mitochondria to the nucleus where it is activated by an apoptotic agent to cause apoptosis. It has abnormal expression in a variety of tumor tissues, including colorectal, lung, breast, pancreatic, renal cell, and other cancers (Xiong et al. 2016; Zong and Liang 2023). Rao and colleagues have determined that down-regulating AIF expression within a physiological context without apoptotic stimulation inhibits mitochondrial aerobic respiration and encourages the transition of ATP synthesis to the glycolysis pathway. This condition has been shown to be consistent with the “Warburg effect (Rao et al. 2019). AIF is known as a trigger of cell death and is required in some stimulus-induced apoptotic pathways (Agarwal et al. 2014).

Drug resistance remains one of the major barriers to effective treatment for advanced colorectal cancer, and understanding the underlying mechanisms of this phenomenon is limited.

The purpose of this study is to ascertain how capecitabine-resistant HT29 CRC cell lines respond to fisetin, capecitabine, and combinations of the two medications. Combined treatment of fisetin and capecitabine is thought to contribute to increased cell proliferation and inhibition of apoptosis in capecitabine-resistant HT29 CRC cells compared to wild-type HT29 cells. The molecular mechanism by which fisetin definitively increases CRC cell susceptibility to chemotherapeutic agents that function through different mechanisms is currently unknown; however, our study will lay the groundwork for cell signal transduction pathways and protein-gene expression studies. In addition to supporting the limited publications in the literature, our study will contribute to the pathophysiology of CRC, the analysis of the resistance mechanism, and the combination of flavonoids with chemotherapy drugs. The trial of new drug combinations will also provide a different perspective on CRC research and countering drug resistance. This research study will contribute to the increase of our scientific competition in cancer research.

## Materials and methods

### Cell culture and reagents

We bought the HT29 human colorectal cancer cell line from ThermoFisher (USA). The cell lines were cultured in high-glucose Dulbecco’s Modified Eagle Medium (DMEM) (Capricorn, Germany) supplemented with 10% fetal bovine serum (FBS) (Capricorn, Germany) and 1% penicillin/streptomycin, and they were cultured at 5% CO_2_ and 37 °C. Capecitabine was commercially purchased from (Selleck Chemicals GmbH, S1156, Planegg, Germany). Fisetin was purchased from Glentham Life Sciences Ltd (GL7384, Glentham, United Kingdom).

### Establishment of capesitabine-resistant HT29 cell lines (CR/HT29)

Wild-type HT29 cell lines were incubated in DMEM culture medium containing FBS with 1% penicillin/streptomycin at 37 °C with 5% CO_2_. Resistant cell lines were generated with minor modifications to the previously described method (Jiang et al. 2013; Zhou et al. 2020). First adaptation step: Wild-type HT29 cells were treated with capecitabine for 48 h with gradually increasing drug concentration (1, 5, 10, 20, and 40µM). The medium was changed every 3 days and the drug exposure dose increased every 15 days. After each dose-dependent step, apoptotic cells were discarded, and surviving cells were grown in culture medium without capecitabine. This step was repeated three times. Cells were then exposed to the next dose of capecitabine. In the second step, consolidation, cells were treated with an ultimate concentration of capecitabine (40µM), thereby generating capecitabine (CR/HT29) resistant cells. These CR/HT29-resistant cells were continued to be grown in a DMEM medium. Furthermore, wild-type HT29 and CR/HT29 cells were viewed at ten-fold magnification using an inverted microscope (Olympus, Tokyo, Japan).

### Reverse transcription-polymerase chain reaction (RT-PCR)

Total RNA was extracted from cells (< 2 × 10^6^ cells) using Direct-zol™ RNA Miniprep Plus kit (ZYMO, USA). The cDNA synthesis was carried out by taking 8 µL of the RNA stock solution in accordance with the manufacturer’s instructions. The reaction content consists of 10 µL of 2X miRNA cDNA Synthesis SuperMix, 2 µL of Enzyme Mix, and 8 µL of RNA template. The prepared mixture was incubated at 37 °C for 30 min, at 50 °C for 15 min, at 85 °C for 5 min, and then at 4 °C. The obtained cDNA sample was stored at −20 °C to be used in mRNA analysis. RT-PCR was performed using SensiFast SYBR No-ROX kit (Bioline, USA) on a Roche LightCycler 480 system using primers specifically designed for P-gp and GAPDH to be used as a reference. Primers were designed using miRprimer software and primer sequences are presented in Table 1. The cDNA amplification was performed with an initial activation step for 5 min at 95 °C followed by a 10 s denaturation at 95 °C and 60 s binding/elongation cycle at 60 °C, repeated 45 times. Each sample was examined in duplicate, and all data were analyzed using the 2^−ΔΔCT^ method (Pfaffl, 2001). Statistical analysis was carried out using SPSS 19.0 (SPSS, Chicago, IL, USA) and GraphPad Prism 8 (GraphPad, La Jolla, CA, USA). All tests were performed using a two-sided test and non-parametric tests were used to compare groups. Factors with *P* < 0.05 were considered statistically significant.

**Table 1:** mRNA primer sequences

Primer	Sekans
P-glicoprotein (P-gp) forward	5 ′-GCCTTCATCGAGTCACTGCC-3 ′
P glicoprotein (P-gp) reverse	5 ′-CCAGGGCTTCTTGGACAACC-3 ′
GAPDH forward	5 ′-GGTCACCAGGGCTGCTTTTA-3 ′
GAPDH reverse	5 ′-CCCGTTCTCAGCCATGTAGT-3 ′

Additionally, the RT-PCR conditions used for the detection of P-gP mRNA expression levels are listed in Table 2.

**Table 2:** RT-PCR conditions for the detection of P-gP expression levels

Step	Time	Temperature (°C)
PCR initial activation	5 min	95 °C
Denaturation	10 s	95 °C
Annealing/extension	60 s	60 °C
Number of cycles	45 cycles	

### BrdU (5-bromo-2′-deoxyuridine) cell proliferation assay

The trypan blue exclusion test and cell counter (TC-20 BioRad, Hercules, CA, USA) were used to evaluate cell viability tests, and the BrdU cell proliferation assay was used to evaluate cell proliferation investigations. Wild-type HT29 cells (2 × 10^4^) and CR/HT29 cells (2 × 10^4^) were seeded into 96-well plates containing serum-free culture media. After 24 h, the “starved cells” were transferred to DMEM medium supplemented with 1% FBS at 37 °C under with 5% CO_2_ conditions. Both cell lines were incubated for 24 h with fisetin (60µM, 90µM, 120µM), capecitabine (40µM), and/or combination of these drugs. Cells were harvested, and cell proliferation was evaluated following the manufacturer’s instructions using the BrdU Labeling and Detection Kit (Roche, Mannheim, Germany). Samples were measured using a Biotek Synergy H1 instrument (Synergy H1, BioTek, United States) at a wavelength of 370 nm.

### Morphological assessment of cells by hoechst staining

The cells were incubated in a carbon dioxide incubator for 24 h, after which the medium was discarded, and the wells were washed with PBS. After being fixed for 10 to 15 min at room temperature with 4% paraformaldehyde, wild-type HT29 and CR/HT29 cells were PBS-washed three times. Then fixation with 70% ethanol and subsequent PBS washes, the cells were stained with Hoechst (Invitrogen, Thermofisher Scientific, USA) dye (10 mg/mL) dissolved in PBS for 3 min at room temperature under dark conditions. Following staining, the cells were washed again with PBS. Cell images were acquired using a confocal microscope (Nikon, Ti Eclipse, Amsterdam, Netherlands) at twenty-fold magnification to observe morphological changes (White et al. 2003).

### Scratch-wound assay

The wound healing experiment was conducted to observe the migration abilities of wild-type HT-29 and CR/HT29 cells. Cells were seeded into a 12-well plate at a density of 50 × 10^3 cells per well, and incubated until reaching 90% confluency in each well. Using a 200 µl pipette tip, the cell layer covering the plate was scratched, and any cell debris was then removed by washing with PBS (Grada et al. 2017). After the application of drugs singly and in combination for 24 h, the medium was discarded, and fresh medium was added immediately (0th hour). Images were captured at 0, 24, and 48 h using an inverted microscope (Olympus, Tokyo, Japan). All samples were analyzed in triplicates. With Image J software, the degree of wound healing was evaluated (National Institutes of Health, Bethesda, MD, USA). Analyses were conducted in triplicates.

### Colony formation assay

To assess the colony-forming capacity of wild-type HT29 and CR/HT29 cell lines, cells were seeded at a density of 1 × 10^3^ cells/well in 6-well plates, and cells were incubated at 37 °C in a carbondioxide incubator. The cells were drug-treated for 24 h following seeding. The medium was discarded after 24 h, the cells were cleaned with PBS, and new medium was introduced. Throughout the incubation period, the medium was refreshed, and cells were monitored for colony formation for 11 days. After colonies formed, cells were fixed and stained with 1% crystal violet (Fu et al. 2018). The analysis was performed in triplicate. ImageJ software (National Institutes of Health, Bethesda, MD, USA) was used to evaluate colony counting. The percentage of cell colony formation was determined using the following formula:

$$\mathrm{Colony}\;\mathrm{formation}\;\mathrm{effiency}\left(\%\right)=\frac{\mathrm{Number}\;\mathrm{of}\;\mathrm{colonies}\;\mathrm{formed}}{\mathrm{Number}\;\mathrm{of}\;\mathrm{cells}\;\mathrm{planted}\left(10^3\right)}\times100\%$$

### Western blot analysis

Drugs were administered to CR/HT29 and wild-type HT29 cells for a whole day in DMEM media containing 1% serum. Following cell harvesting and a 15-min PBS wash at 300 g, the pellets were frozen at −80 °C. The resulting pellets were resuspended in lysis tampon containing a Mini Protease Inhibitor Cocktail (Roche, Merck, Germany). With the use of the BCA protein assay kit (Thermo Scientific, USA), the protein content of the whole lysates was assessed (Lowry et al. 1951). Proteins were separated into equal parts (50 µg/lane) on 10% SDS-PAGE gels before being moved to nitrocellulose membrane. The membranes were incubated with cas-3, BcL-2, Bax, cyt c (Invitrogen, UK), apoptosis-Inducing Factor (AIF), cas-9 (Cell signalling, Massachusetts, ABD), and actin and cas-8 (SantaCruz, USA) primary antibodies respectively. The membranes were treated with goat anti-rabbit secondary antibodies coupled with alkaline phosphatase and horseradish peroxidase after being washed with TBS containing 0.05% Tween-20 (TBS-T). This was followed by enhanced chemiluminescent labeling with the BCIP/NBT and ECL system (İnvitrogen, UK). Western blot analyses were performed with only modest changes from our earlier research (Lowry et al. 1951). Optical density was used to measure band intensities using ImageJ’s free edition. For every blot, actin served as a housekeeping gene control. Cas-3, BcL-2, Bax, cyt c, AIF, cas-9, cas-8, and actin have apparent molecular weights of 32 kDa, 26 kDa, 23 kDa, 15 kDa, 67 kDa, 37 kDa, 18 kDa, and 43 kDa, in that order.

### Statistical analysis

The Standard Error of the Mean (±SEM) of three or more experiments is used to express the data. Tukey’s post-tests were used after the two-way variance analysis to determine all statistical analyses. Tukey’s multiple comparisons test was used for all statistical tests, which were carried out using GraphPad Software Inc.’s Prism version.

## Results

### Establishment of capecitabine-resistance in wild-type HT29 cell lines

**Fig. 1:**
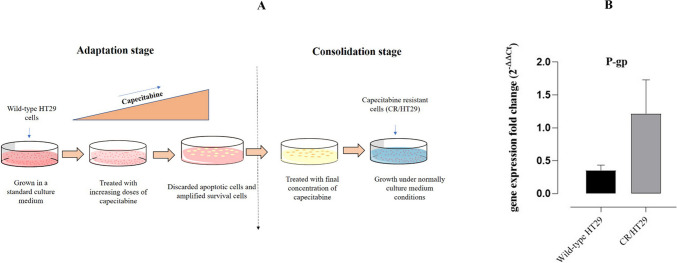
**A** Schematic diagram showing the generation of capecitabine-resistant cells in the wild-type HT29 cell line. (adapted from Zhou et al. 2020). **B** Graph of P-gp expression levels in wild-type HT29 and CR/HT29 cells. After RNA extraction from wild-type and resistant cells, cDNA synthesis was performed using appropriate primers. Real-time PCR (RT-PCR) was conducted. GADPH was used as the housekeeping gene

Briefly, to develop drug resistance, the method consists of two steps. In the adaptation phase, wild-type HT29 cells were exposed to capecitabine at progressively higher doses (1µM, 5µM, 10µM, 20µM, and 40µM) for a duration of 48 h. Apoptotic cells were disposed of after 48 h, and new medium was supplied along with a modification in the cell medium. By the way passage of cells a few times, and surviving cells were grown in culture medium without capecitabine the next dose was passed and each dose step was repeated three times. In the consolidation step, the cells were subject to the final dose of capecitabine for 48 h, the cell medium was refreshed after 48 h. Then, HT29 cells continued to be grown in normal cell medium without capecitabine. After 3–6 months of treatment, CR/HT29 were established (Mohammadian et al. 2017, 2019; Sim et al. 2018; Zhou et al. 2020) (Fig. 1A). P-gp expression was shown in Fig. 1B.

### Fisetin markedly inhibited cell proliferation of colorectal cancer cells and its combination with capecitabine exhibited synergistic effects

**Fig. 2:**
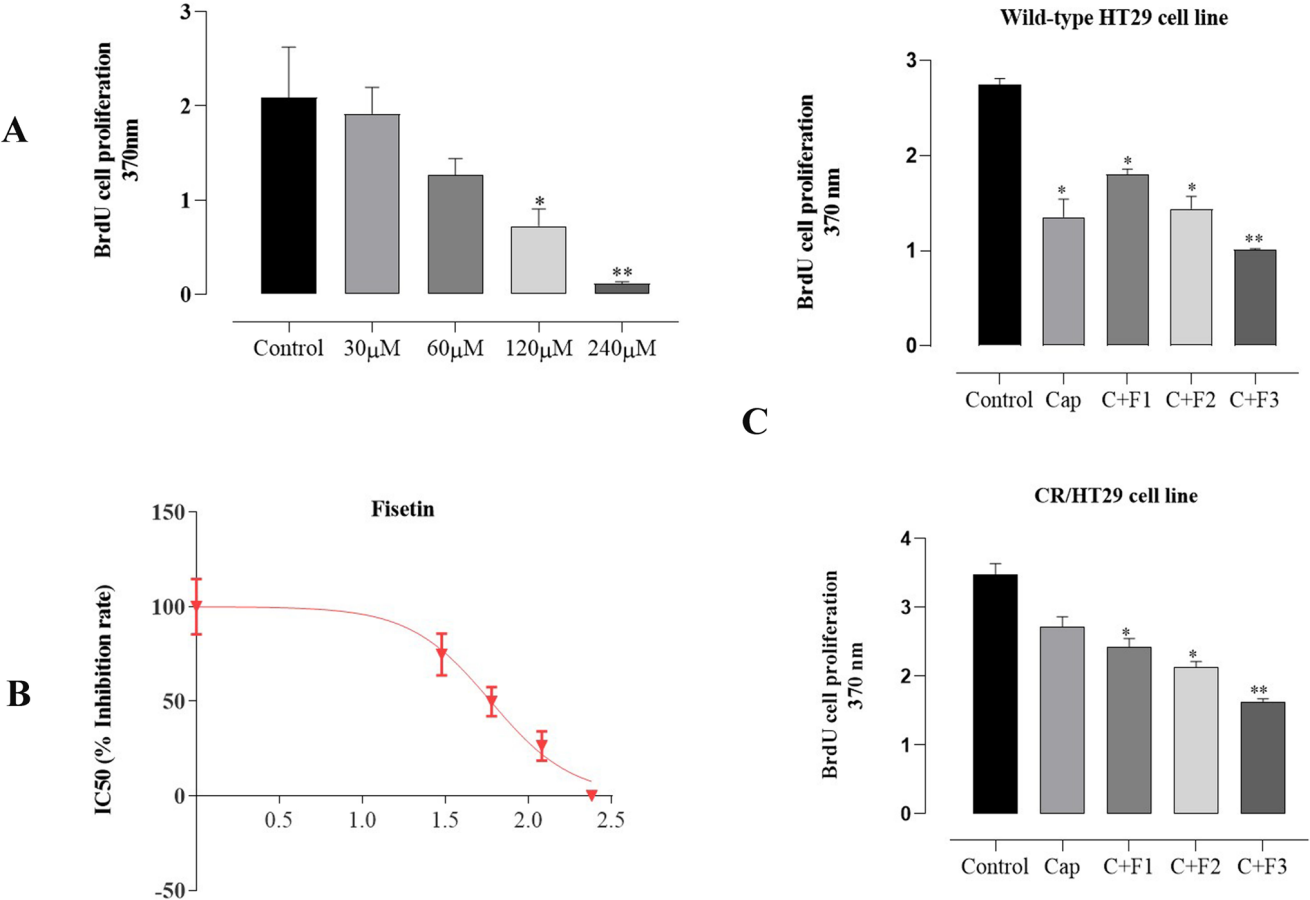
The BrdU-assay shows the effect of flavonoids on cell proliferation (%) of wild-type HT29 and CR/HT29 cells. Fisetin decreased the resistance of the cells to chemotherapeutic drugs. Cells were incubated for 24 h with fisetin (60, 90, 120µM) and capecitabine (40µM). **A** Dose-response curve of fisetin. **B** IC_50_ value of fisetin in wild-type HT29 cells. **C** Effect of drugs on cell proliferation individually in wild-type HT29 and CR/HT29 cells after treatment with concentrations of capecitabine and/or fisetin for 24 h (*p < 0.037716; **p < 0.002791). The treated cells were evaluated against the control group. All experiments were conducted in quintuplicates, and the data were expressed as the mean ± SEM (standard error) (*n* = 5) (Abbreviations: Cap; capecitabine, C; 40µM capecitabine, F1; 60 µM fisetin, F2; 90µM fisetin, F3; 120µM fisetin)

We first performed BrdU assay on wild-type HT29 cells. To investigate the inhibitory effect of fisetin on wild-type HT29 and CR/HT29 cells, the proliferation in two cell lines was evaluated with the BrdU assay after treating them with only capecitabine, only fisetin, and simultaneously, both of them for 24 h. Cell proliferation was detected according to the manufacturer’s protocol (Roche BrdU kit, Germany). Fisetin dose-response experiments were made to determine an effective dose. Treatment of wild-type HT29 cells with fisetin 120µM and 240 µM inhibited cell proliferation compared to control (*p < 0.019; **p < 0.003, respectively (Fig. 2A). The IC_50_ value of fisetin in wild-type HT29 cell proliferation experiments was determined as 59.13µM in the dose-response curve, (Fig. 2B) and 60µM, 90µM, and 120µM fisetin doses were used in wild-type HT29 and CR/HT29 cell lines in cell proliferation experiments. The incubation of wild-type HT29 and CR/HT29 cell lines with different concentrations of fisetin (60µM, 90µM, and 120µM), 40µM capecitabine and /or combinations of drugs produced important alterations in the cell proliferation.

During the 24-h incubation periods, proliferation tests were conducted on both wild-type HT29 and CR/HT29 cells. The chemotherapy drug capecitabine alone or combined with fisetin showed a similar effect on cell proliferation at 24 h in wild-type HT29 cells. However, a difference was seen in CR/HT29 cell lines when capecitabine alone was compared with the capecitabine-fisetin combination (Fig. 2C ).

Accordingly, the results of the cell proliferation experiment; 40µM-capecitabine, 60µM-, 90µM-, and 120µM-fisetin showed a more significant inhibitory response in wild-type HT29 cells compared to CR/HT29 resistant cells, respectively (****p < 0.0001). 40µM-capecitabine + 60µM-fisetin; 40µM-capecitabine + 90µM-fisetin; 40µM capecitabine + 120 µM fisetin together resulted in a more significant inhibitory response in wild-type HT29 cells compared to CR/HT29 resistant cells respectively (*p < 0.0459; ***p < 0.0008, ****p < 0.0001, respectively). Therefore, capecitabine effects on CR/HT29 cell line less than wild-type HT29 cells. These results confirm the development of capecitabine resistance. Besides, exposure to fisetin and/or capecitabine induced an inhibitory response in both cell lines. These results showed that CR/HT29 cells developed resistance to capecitabine, and it was also determined that fisetin had a more significant inhibitory effect on CR/HT29 cell proliferation 120µM was chosen as the dose of fisetin in the following experiments.

### Fisetin alone inhibited scratch-wound of wild-type HT29 and CR/HT29 cells better than the fisetin and capecitabine combination

**Fig. 3:**
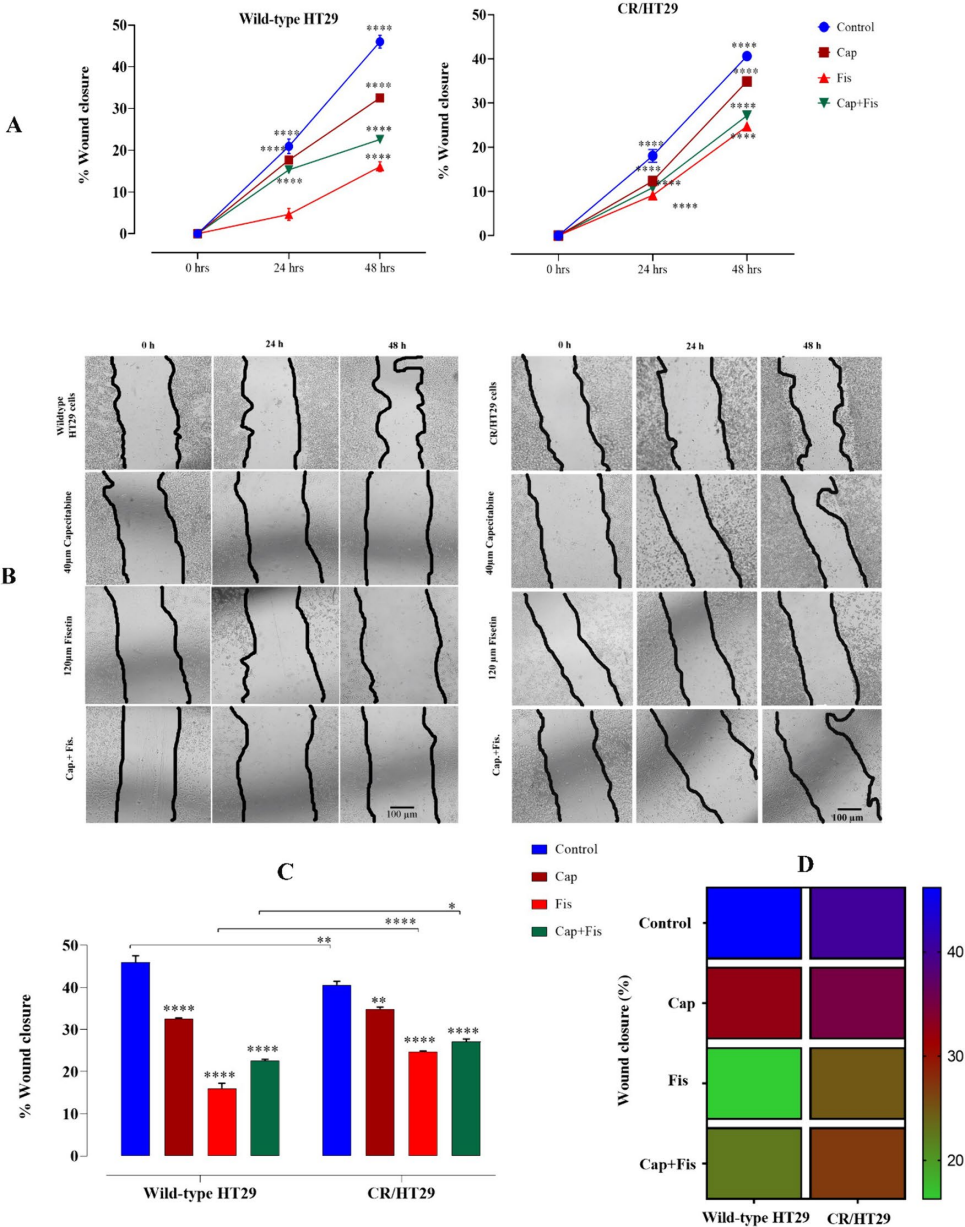
Capecitabine resistance is associated with metastasis in CRC. Scratch-wound assay of wild-type HT29 and CR/HT29cells. **A** Plots of percent wound closure in the wild-type HT29 and CR/HT29 cell line (**** *P* < 0.0001). **B** Sample images of in vitro scratch assay for 0., 24., and 48 h. **C** Wild-type HT29 and CR/HT29 cell line percent wound closure comparison plot at 48 h (*p < 0.0180; **p < 0.0044;****p < 0.0001, respectively). **D** Heatmap representation of wound healing. Data are presented as the mean ± SEM of fewest four experiments

Scratch-wound was determined by performing a scratch assay in the presence of capecitabine and/or fisetin. Phase-contrast images were taken instantly 0 h, 24 h, and 48 h after scratching the plates, and the wound widths were calculated using ImageJ Software. The cells were scratched 0 h and sustained in complete medium for 24 h amd 48 h. The black lines indicate the edges of the wounds. According to the results in cell scratch-wound experiments, cell groups tended to close (heal) in both cell lines, but the control group showed more significant closure at 24 and 48 h compared to other groups. 120µM fisetin group showed less closure in wild-type HT29 cells compared to all other groups ****p < 0.0001. Fisetin inhibits cell proliferation more strongly in wild-type cells (Fig. 3A). Likewise, the wounds tend to close in CR/HT29 cell groups. The change in the percentage of closure value was different from wild-type cell because capecitabine or capecitabine + fisetin was less effective in resistant cells compared to the wild-type HT29 cells (*p < 0.0180;**p < 0.0044; ****p < 0.0001, respectively) (Fig. 3C).

### The combination of fisetin and capecitabine inhibited the colony formation of wild-type HT29 and CR/HT29 cells better than either drug alone

**Fig. 4:**
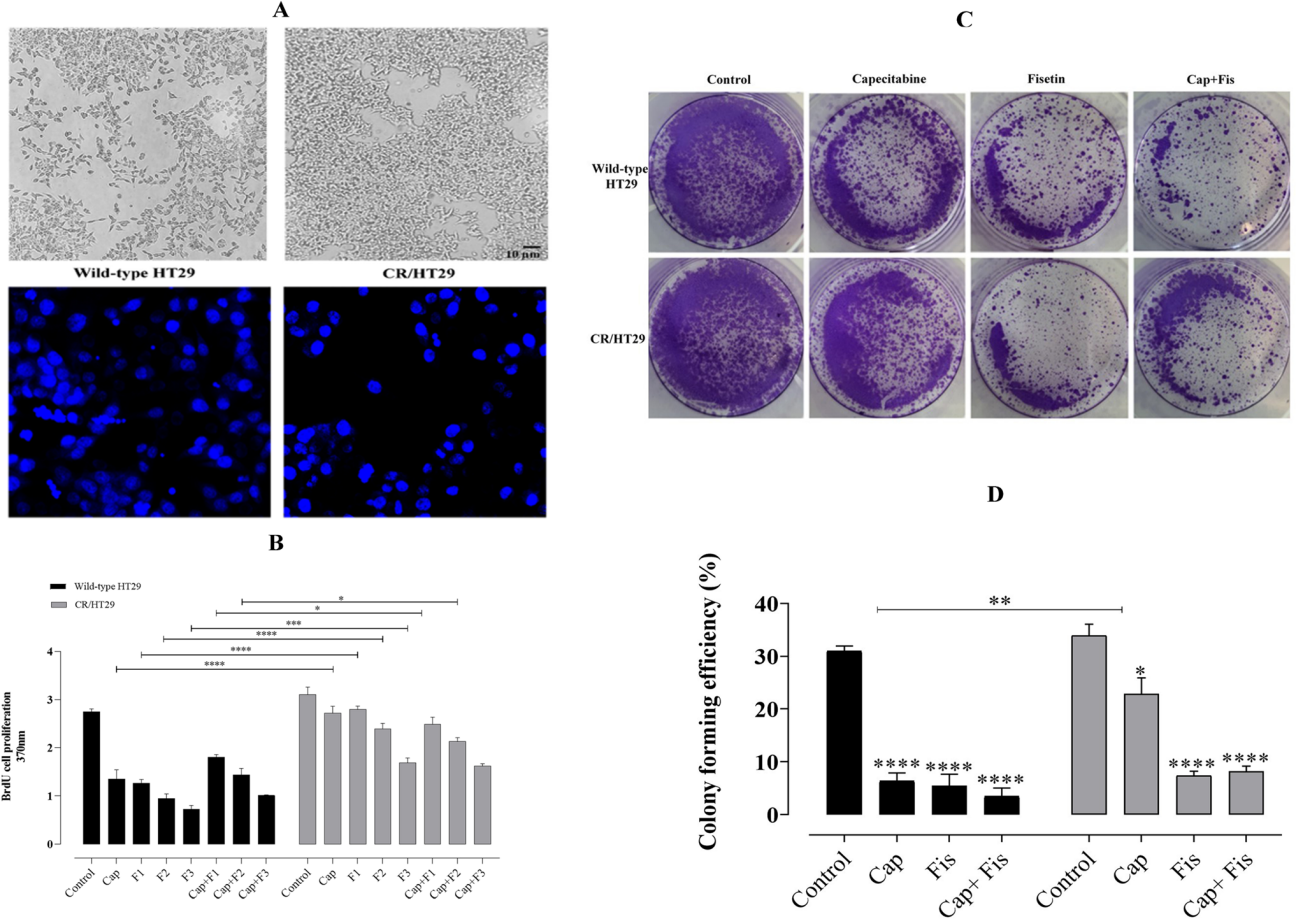
Fisetin and/or capecitabine inhibits colony formation and cell proliferation in wild-type HT29 and CR/HT29 cells. **A** Morphology of wild-type HT29 and CR/HT29 cell lines growing in 2D cell culture condition. Wild-type HT29 and CR/HT-29 cells show significant alteration in cellular morphology (10× magnification) an inverted microscope. Scale bar = 10 μm. Acquired capecitabine-resistant HT29 cells show morphological changes compared with wild-type HT29 cells. Confocal microscopy (Nikon Ti Eclipse, Amsterdam, Netherlands) of HT29 and CR/HT29 cells stained with Hoecsht (20× magnification), **B** Effect of drugs on cell proliferation individually in wild-type HT29 and CR/HT29 cells after treatment with concentrations of capecitabine and/or fisetin for 24 h (*p < 0.037716; **p < 0.002791). The treated cells were evaluated against the control group. All experiments were conducted in quintuplicates, and the data were expressed as the mean ± SEM (standard error) (*n* = 5). **C** The representative images show colony formation assay in wild-type HT29 and CR/HT29 cells. Data are expressed as a percentage relative to control cells. Data are presented as the mean ± SEM of fewest four experiments. **D** Quantitative analysis results of colony formation efficiency of wild-type HT29 and CR/HT29 cells (*p < 0.0147; **p < 0.0041; ****p < 0.0001)(Abbreviations: Cap; capecitabine, C; 40µM capecitabine, F1; 60 µM fisetin, F2; 90µM fisetin, F3; 120µM fisetin)

We investigated whether combination drug treatments inhibit colony-forming ability, an essential process in colorectal cancer cell growth. According to the calculation made with the colony formation efficiency formula, the following results were obtained (Fig. 4). Both wild-type HT29 and CR/HT29 cells are inhibited by exposure to fisetin and/or capecitabine. As shown in Fig. 4A and C, when HT29 wild-type cells were compared with CR/HT29 cells, cell morphology was altered and showed an aggressive phenotype. Morphological and biological changes are generally thought to be associated with drug resistance. Effects of drugs on cell proliferation were shown in Fig. 4B. The combination of capecitabine and fisetin demonstrated the most reducing effects on colony formation compared to the control group in wild-type HT29 cells. In contrast, fisetin alone showed a more inhibitory effect than the control group in CR/HT29 cells (****p < 0.0001) (Fig. 4D). The percentage of colony formation in the 40µM capecitabine CR/HT29 cells was higher than in wild-type HT29 cells (**p < 0.0041). In CR/HT29 cells, a dose of 40µM capecitabine inhibited colony formation slightly compared to the control group (*p < 0.0147). Compared to the control group, the combination of capecitabine and fisetin in CR/HT29 cells significantly inhibited colony formation ( ****p < 0.0001). Colony formation results were found to be in agreement with the results obtained from cell proliferation and scratch wound experiments.

### Effects of drugs on apoptosis protein expression in wild-type HT29 and CR/HT29 cancer cells

**Fig. 5:**
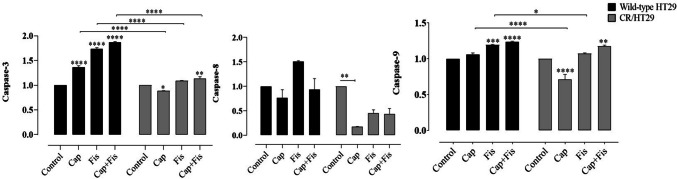
Expressions of cas-3, cas-8, and cas-9 in wild-type and CR/HT29 cells. Densitometric analysis data shown in graphThe data are expressed as the means ± SEM of fewest three experiments when compared to control (*p < 0.0323; ** *p* < 0.0017 ; ***p < 0.0005; ****p < 0.0001, respectively)

The expression of apoptosis proteins in cells was then measured in order to investigate a possible molecular basis for fisetin-mediated cell death. Western blot analysis was performed to see if capecitabine and/or fisetin would induce apoptosis in wild-type HT29 and CR/HT29 cancer cells. Expressions of cas-3, cas-8, and cas-9 expression were shown in Fig. 5.

**Fig. 6:**
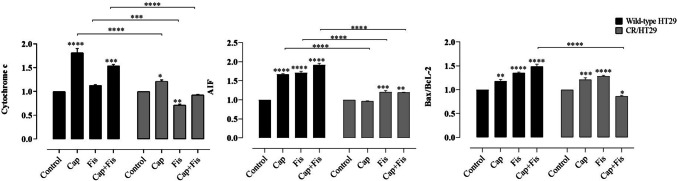
The effect of capecitabine and/or fisetin on the expression of cytochrome c, Bax/ BcL-2, and AIF expression in wild-type and CR/HT29 cells. Densitometric analysis data shown in graph, The data are expressed as the means ± SEM of fewest three experiments when compared to control (*p  < 0.0323; **p  < 0.0017; ***p  < 0.0005; ****p  < 0.0001, respectively)

Our results have shown that all apoptosis protein levels significantly increased in wild-type HT29 cells in fisetin and/or capecitabine combinations but much less affected CR/HT29 cells. When both groups were compared, cas-3 expression significantly increased when all drugs were applied together in wild-type HT29 cells, while in CR/HT29 cells, cas-3 levels markedly decreased (****p < 0.0001). Because CR/HT29 cells have developed a resistance mechanism to capecitabine. In addition, cas-3 expression in resistant CR/HT29 cells was significantly increased in fisetin combined with capecitabine compared to control group (**p < 0.0056). When comparing the wild-type HT29 and CR/HT29 cell lines, cas-3 expression was inhibited in CR/HT29 cells compared to wild-type HT29 cells at all drug treatments (****p < 0.0001) (Fig. 5). In wild-type HT29 cells, cas-9 levels increased in all drug groups (****p < 0.0003), while capecitabine decreased cas-9 levels in CR/HT29 cells (****p < 0.0001). In CR/HT29 cells, capecitabine significantly increased cas-9 when co-administered with fisetin (**p < 0.0011). When capecitabine groups were compared in these two cells, cas-9 expression was inhibited in CR/HT29 cells relative to wild-type HT29 cells (****p < 0.0001) (Fig. 5). In CR/HT29 cells, capecitabine significantly reduced the levels of cas-8 compared to control (**p < 0.0029). In the fisetin and capecitabine combination treatment groups, there was a non-significant decrease as well. In the wild-type HT29 cell group, fisetin caused a insignificant enhance in cas-8 levels compared to the control group. When these wild-type and resistant cell groups were compared, it was seen that CR/HT29 cells inhibited cas-8 levels in all drugs (*p *> 0.005). AIF levels were increased in wild-type HT29 cells compared to control in all drug groups. AIF levels were considerably enhanced in CR/HT29 cells when capecitabine was administered alone or in combination with fisetin compared to control groups (** *p* < 0.0017; ***p < 0.0005). When these two cell groups are compared, it is seen that CR/HT29 cells inhibit AIF expression in all drug groups compared to wild-type HT29 cells (****p < 0.0001) (Fig. 6).

Fisetin-only, capecitabine-only and capecitabine + fisetin compared with control caused increased Bax/BcL-2 protein expression in wild-type HT29 cells (**p < 0.0018; ****p < 0.0001). In CR/HT29 cells, while only-capecitabine and only-fisetin caused an increase compared to the control group, When capecitabine and fisetin were administered together in the CR/HT29 cells, it caused inhibition of Bax/BcL-2. (*p < 0.0164; ***p < 0.0004; ****p < 0.0001 respectively). When wild-type HT 29 and CR/HT29 cells treated with capecitabine and fisetin were compared, Bax/BcL-2 protein level was inhibited in CR/HT29 cells compared to wild-type HT29 cells (****p < 0.0001) (Fig. 6).

In the wild-type HT29 cells, capecitabine treatment increased cytochrome c levels (****p < 0.0001). When applied together, capecitabine and fisetin gave rise to an increase in cytochrome c levels compared to the control group (***p < 0.0003). Capecitabine increased cytochrome c levels by 21% but fisetin caused a 29% inhibition in CR/HT29 resistance cells. When these wild-type and CR/HT29 cell groups were compared, cytochrome c levels were found to be inhibited in all drug groups in CR/HT29 cells (***p < 0.0005 ; ****p < 0.0001) (Fig. 6).

**Fig. 7:**
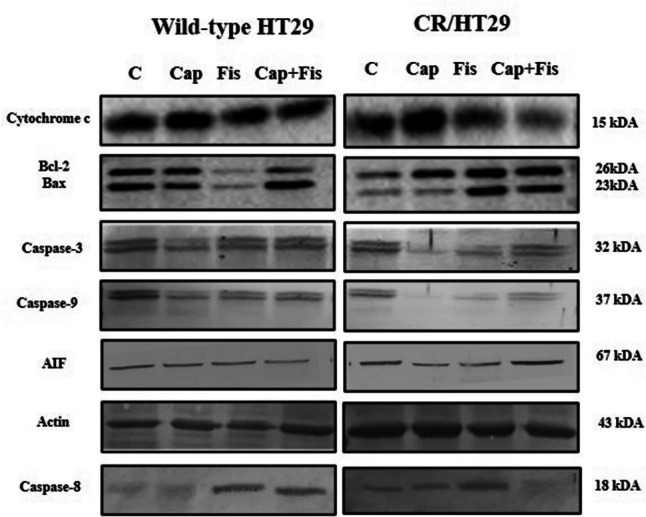
Expressions of cytochrome c, BcL-2, Bax, cas-3, cas-9, cas-8, and AIF expression in wild-type and CR/HT29 cells cells. Protein expression are analyzed by western blotting. The representative images of nitrocellulose membranes were attained from western blot analyses exhibiting protein expressions of cas-3, cas-9, cas-8, BcL-2, Bax, cytochrome c, and AIF in the wild-type HT29 and CR/HT29 cells

The representative images of western blots are shown in (Fig. 7).

The decrease in cas-3, cas-9, AIF, and cytochrome c levels in CR/HT29 cells compared to wild-type HT29 cells indicates that capecitabine resistance has developed. As a result, the cells are partially protected from undergoing apoptosis.

## Discussion

According to our study, we demonstrated that fisetin induces apoptosis in wild-type HT29 cells and partly inhibits the apoptotic signaling pathway involved in CR/HT29 cell proliferation. We established the capecitabine-resistant cell subset CR/HT29 in wild-type HT29 cells by employing a dose escalation strategy through continuous exposure to capecitabine. According to our results, the development of resistant cells is achievable through continuous exposure to increasing doses of capecitabine (Fig. 1A).

Different researchers were determined that the upregulation of P-gp was effective in escaping chemotherapy-mediated cell death in cancer cells (Ambudkar et al. 2003). In our study, the demonstration of the increase in P-gp mRNA expression level in capacitabine-resistant HT29 cells by Real-Time Polymerase Chain Reaction showed that the capecitabine chemo-resistance mechanism is associated with increased intracellular P-gp expression in resistant colorectal cancer cells. Recent studies have focused on developing natural compounds with chemopreventive activities (Afzal et al. 2023; Syed et al. 2008). Co-treatment of anti-tumor agents with flavonoids is a potential target to improve the way chemotherapy affects cancer cells (Lin et al. 2016). Data in this study demonstrated that treatment of wild-type HT29 cells with 120µM and 240µM fisetin had an inhibitory effect on the dose-response curve in the cell proliferation. The IC_50_ value of fisetin in wild-type HT29 cell proliferation experiments was determined as 59.13µM in the dose-response curve (Fig. 2B). Jeng et al. showed that fisetin (80µM) triggered apoptosis in OR and CPT11-LoVo cancer cells at rates of 9.8%, 4%, and 40.3%, respectively (Jeng et al. 2018). It was reported by Suh et al. that both cell lines, HCT116 and HT29 cells, were vulnerable to fisetin and inhibited the proliferation of colon cancer cells (Suh et al. 2009).

In Sim et al. research, a 20µM dose of capecitabine caused inhibition in HT29 cells (Sim et al. 2018). Minghai et al. suggested that cell proliferation of HT29 cells was considerably decreased compared to their respective negative controls after treatment with capecitabine or transfection with si-RANK (Shao et al. 2022). It was proposed by Mohammadian et al. that in a dose-dependent study in which HT29 and HCT-16 cell lines were exposed to capecitabine at different concentrations (1, 2, 4, and 8µM). Capacitabine significantly increased the inhibitory effect of cell viability compared to control cells (Mohammadian et al. 2017). Khan et al. showed that the 90µM fisetin and combination with 5-FU had the greatest inhibitory effect on PIK3CA-mutant HCT116 and HT29 colon cancer cells (Khan et al. 2019). In our investigation, it was determined that the 40µM capecitabine dose, which we decided by examining the literature, caused significant inhibition in both CR/HT29 and wild-type HT29 cells (Fig. 2C and D). When both groups were compared to each other, capecitabine exposure appeared to produce less inhibition in capecitabine-resistant CR/HT29 cells than in wild-type HT29 cells (Fig. 2C and D). Wild-type HT29 cells began to die after capecitabine treatment, but resistant cells survived for a long time. Wu et al. suggested that human DLD-1 CRC cells treated with a combination of capecitabine and visfatin had improved cell proliferation compared to cells treated with capecitabine (Wu et al. 2021). These insights from the literature are consistent with our observations and highlight the potential impact of combination treatments in influencing cell behavior. Zeynali-Moghaddarn et al. demonstrated that the proliferation of HT29 cells decreased in a dose-dependent manner 24 h after treatment with capecitabine, irinotecan, and 17-AAG. Additionally, it was emphasized *t*hat the triple combination group (17-AAG/Cap/irinotecan) showed a definite antagonistic effect, but the double combination groups of 17-AAG, irinotecan, or capecitabine showed synergistic effects in study (Zeynali-Moghaddarn et al. 2019). On the other hand, Kim et al. determined the anticancer effects of capecitabine for clinical application of combined capecitabine therapy with trifluridine/tipiracil or trifluridine/tipiracil in both capecitabine-sensitive COLO205, HCT116 and LOVO cells and capecitabine-resistant HT29 and RKO xenografts (Kim et al. 2017). The results of the present study, 120µM fisetin group showed the highest inhibitory effect on wild-type HT29 cells when compared to all groups (Fig. 2C). It demonstrated also that 120µM fisetin more inhibitory effect when compared to control group on CR/HT29 cells proliferation, however, in wild-type HT29 cells, 120µM fisetin had a greater inhibitory effect compared to CR/HT29 cells. The reducing effect of fisetin showed that resistance could be partially overcome in resistant cells.

Cell migration assays are indicators of metastasis; thus, the invasive talent of HT29 cells in due course of capecitabine-resistance acquisition was evaluated via wound healing assay. In wound healing experiments, both cell lines showed a decrease wound healing in fisetin and fisetin plus capecitabine-treated groups (Fig. 3). In other words, in the groups with fisetin and/or capecitabine, a greater decrease was observed in wild-type HT29 cells than in CR/HT29 cells. Thus, it shows the relationship of fisetin with apoptosis in reducing wild-type HT29 cells. However, the repair process took considerably longer in samples treated with capecitabine and fisetin single or together. Samples receiving the co-treatment experienced larger scratched area and underwent a slower healing process, confirming a reduction in the migration ability of capecitabine-resistant HT29 cells. Also, colony formation assay was conducted to investigate whether there was an effect on the ability of cells to form colonies during the acquisition of capecitabine-resistance. At day 11, the mean colony count of cells decreased with fisetin and/or capecitabine treatment in both wild-type HT29 cells and CR/HT29 cells (Fig. 4). Therefore, it is possible to say that fisetin has a potential to help reduce resistance in the CR/HT29 cell line. In addition, in our study, it was confirmed that the cells showed some nuclear morphological changes together with Hoechts staining in wild-type HT29 and CR/HT29 cell lines (Fig. 4). Cheng-Fang Tsai et al. demonstrated that as the dose of fisetin increased, it decreased cell migration in both 4T1 and JC breast cancer cells compared to the control, and significantly inhibited colony formation ability in both 4T1 and JC breast cancer cells (Tsai et al. 2018). In the study conducted by Sim and colleagues, when the single and co-treatment effects of lactate calcium salt (LCS) and capecitabine on colorectal cancer cell colony formation were compared, it was reported that the combination of LCS and capecitabine resulted in less colony formation compared to single treatments, and the combined treatment significantly reduced the number of HT29 and HCT116 colonies (Sim et al. 2018). Hosseini et al. suggested that combined treatment of quercetin and fisetin in MCF7 and MDA-MB-231 cell lines could significantly reduce the number and forms of colonies in both MCF7 cells and MDA-MB-231 cells. Additionally, improvements in the effect of quercetin and fisetin on the migration of cancer cells were observed in MCF7 after approximately 24 h and in MDA-MB-231 cells after 48 h, depending on the size of the scratched area (Hosseini et al. 2023). In another study, researchers found that fisetin or 5-FU treatment led to a reduction in colony count in both HCT116 and HT29 colon cancer, with a sharper reduction detected with the combination of fisetin and 5-FU (Khan et al. 2019).

According to some theories put forward in the recent decade, it has been shown that fisetin may enhance the anti-tumor effect of chemotherapy drugs by stimulatıng cell cycle arrest and cell death in triple-negative breast cancer cells (Smith et al. 2016). Apoptosis resistance is not only critical for the survival of cancer cells, but also contributes to cancer cells developing resistance to drugs. In studies based on this situation, stimulation of cell death has become an indispensable mechanism of cancer chemotherapy (Suh et al. 2009). In the study by Jeng et al. it was found that treatment of LoVo colon cancer cells with fisetin slightly increased cyt c release, induced cas-8 activation, and increased cleaved cas-3 expression, leading to apoptosis and cell death. Researchers stated that this result is based on different behaviors or properties between various resistant cancer cells (Jeng et al. 2018). According to recent research, AIF improves cancer patient survival, and AIF expression levels are correlated with particular tumor activities. Additionally, overexpression of AIF has been shown to protect malignant colorectal cancer cells from apoptosis under stress (Urbano et al. 2005). In our study, while cas-3, cas-9, AIF, Bax/BcL-2, and cyt c expression levels increased in wild-type HT29 cells in the fisetin and/or capecitabine groups, cas-8 expression increased only in the fisetin group compared to the control. When CR/HT29 cells were compared with wild-type HT29 cells, it was observed that cas-3, cas-8, AIF, Bax/BcL-2, and cyt c protein levels decreased, while cas-9 increased. In CR/HT29 cells, capecitabine slightly increased cyt c levels, but fisetin significantly inhibited it however the two cell lines were compared, it was observed that cyt c expression levels of CR/HT29 cells were inhibited in all drug groups. In other words, the decrease in caspase-3, caspase-8, caspase-9, AIF, and cyt c levels of CR/HT29 cells compared to wild-type HT29 cells indicates that capecitabine resistance has developed.

As a result, it appears that CR/HT29 cells are partially protected from apoptosis. The additive effects of fisetin on the apoptotic pathway of capecitabine in both wild-type HT29 cells and CR/HT29 cells were also seen in wound healing, colony formation, and cell proliferation assays.

The data obtained from our study have provided a newer perspective toward illuminating the molecular mechanisms guiding the identification of new mechanism-based strategies and treatment regimens for cancer prevention and treatment. In conclusion, given the broad chemosensitivity of colorectal cancer to common drug resistance mechanisms, novel approaches are required to enhance response rates, potentially prolong survival, and improve the efficacy of chemotherapy.

## Data Availability

No datasets were generated or analysed during the current study.

## Abbreviations

5-FU: 5-Fluorourasil
5′-DFCR: 5-Deoxy-5-fluorocytidine
AIF: Apoptoz-indükleyici Faktör
BrdU: Bromodeoksiuridin
CRC: Colorectal cancer
cty c: Cytochrome c
5′-DFUR: Doxyfluridine
DMEM: Dulbecco’s Modified Eagle Medium
FBS: Fetal bovine serum
MDR: Multi-drug resistance
NBT/BCIP: Nitroblutetrazolyum/Bromokloroindol fosfat
P-gp: P-glycoprotein
SDS: Sodyum dodesil sülfat
TP: Thymidine phosphorylase
